# Growth Velocity and Nutritional Status in Children Exposed to Zika Virus during Pregnancy from Amazonas Cohort, Brazil

**DOI:** 10.3390/v15030662

**Published:** 2023-03-01

**Authors:** Lucíola de Fátima Albuquerque de Almeida Peixoto, Marília Rosa Abtibol-Bernardino, Cecilia Victoria Caraballo Guerra, Geruza Alfaia de Oliveira, Beatriz Caroline Soares Chaves, Cristina de Souza Rodrigues, Anny Beatriz Costa Antony de Andrade, Elijane de Fátima Redivo, Salete Sara Alvarez Fernandes, Rodrigo Haruo Otani, Alexandre Vilhena da Silva Neto, Antônio Alcirley da Silva Balieiro, Celso Rômulo Barbosa Cabral, Djane Baia-da-Silva, Márcia da Costa Castilho, Camila Helena Bôtto-Menezes, Maria das Graças Costa Alecrim, Maria do Carmo Leal, Silvana Gomes Benzecry, Flor Ernestina Martinez-Espinosa

**Affiliations:** 1 Postgraduate Program in Tropical Medicine (PPGMT), State University of Amazonas (UEA) in Partnership with the Tropical Medicine Foundation Dr. Heitor Vieira Dourado (FMT-HVD), Manaus 69040-000, Brazil; 2 Department of Maternal and Child Health, Medical School, Federal University of Amazonas, Manaus 69020-160, Brazil; 3 Tropical Medicine Foundation Dr. Heitor Vieira Dourado, Manaus 69040-000, Brazil; 4 Leônidas & Maria Deane Institute, ILMD/FIOCRUZ Amazonia, Manaus 69057-070, Brazil; 5 School of Health Sciences, University of Amazonas State, Manaus 69065-001, Brazil; 6 Faculty of Pharmacy, University Nilton Lins, Manaus 69058-030, Brazil; 7 Medical Course Coordination at Manaus Metropolitan College/FAMETRO, Manaus 69050-000, Brazil; 8 Oswaldo Cruz Institute, FIOCRUZ Rio de Janeiro, Rio de Janeiro 21040-360, Brazil

**Keywords:** Zika virus, arbovirus, congenital Zika virus syndrome, child growth, growth velocity, anthropometry, infant nutrition, non-microcephalic children

## Abstract

The high incidence of Zika virus (ZIKV) infection in the period of 2015–2016 in Brazil may have affected linear height growth velocity (GV) in children exposed in utero to ZIKV. This study describes the growth velocity and nutritional status based on the World Organization (WHO) standards of children exposed to ZIKV during pregnancy and followed up in a tertiary unit, a reference for tropical and infectious diseases in the Amazon. Seventy-one children born between March 2016 and June 2018 were monitored for anthropometric indices: z-score for body mass index (BMI/A); weight (W/A); height (H/A) and head circumference (HC/A); and growth velocity. The mean age at the last assessment was 21.1 months (SD ± 8.93). Four children had congenital microcephaly and severe neurological impairment. The other 67 were non-microcephalic children (60 normocephalic and 7 macrocephalic); of these; 24.2% (16 children) had neurological alterations, and 28.8% (19 children) had altered neuropsychomotor development. Seventeen (24.2%) children had inadequate GV (low growth velocity). The frequencies of low growth among microcephalic and non-microcephalic patients are 25% (1 of 4 children) and 23.9% (16 of 67 children); respectively. Most children had normal BMI/A values during follow-up. Microcephalic patients showed low H/A and HC/A throughout the follow-up, with a significant reduction in the HC/A z-score. Non-microcephalic individuals are within the regular ranges for H/A; HC/A; and W/A, except for the H/A score for boys. This study showed low growth velocity in children with and without microcephaly, highlighting the need for continuous evaluation of all children born to mothers exposed to ZIKV during pregnancy.

## 1. Introduction

The Zika virus (ZIKV) is a flavivirus, identified in 1947, transmitted mainly by the bite of the Aedes mosquito [1]. In February 2016, due to outbreaks in the Pacific and mainly in the Americas, ZIKV became an international public health emergency [2]. In the recent epidemic of 2015–2016, sexual, perinatal, and blood [3,4,5,6] transmission was evidenced, in addition to neurological complications, serious complications compatible with congenital Zika virus syndrome (CZVS), changes in growth and development of children whose mothers were exposed during pregnancy, low birth weight, and even fetal loss [7,8,9,10,11,12,13,14,15].

Diffuse placental injury, trophoblast hyperplasia, fetal necrosis, and loss of embryonic blood vessels associated with ZIKV infection can promote serious adverse outcomes in pregnancy, fetus, and neonate [7,16,17,18,19,20]. High frequencies of neonatal adverse outcomes (low birth weight, microcephaly, stillbirth, and premature birth) were especially associated with ZIKV infection during early pregnancy in a cohort of pregnant women exposed to ZIKV in the Brazilian Amazon (Manaus, Amazonas) during the peak period of ZIKV transmission (47th epidemiological week of 2015 to last epidemiological week of 2016) [21]. Similar outcomes associated with the presence of maternal symptoms of ZIKV infection in the first trimester of pregnancy were also evidenced by Souza et al. [22], which highlights the high frequency of intrauterine growth restriction and babies being born small.

In CZVS, the prevalence of low birth weight is four times higher than in newborns without CZVS [10]. In addition to low birth weight, postnatal growth deficits in children with congenital microcephaly and those who are normocephalic but with some neurological abnormality were evidenced [9]. Compromised growth and development in CZVS may be associated with hyperirritability, hyperexcitability, and sucking and swallowing difficulties, which are relevant nutritional findings [23]. However, it is important to highlight that children without microcephaly, but exposed to ZIKV during pregnancy, are smaller, have lower weight, and have a lower percentage of fat compared to non-exposed children [24]. Regardless of microcephaly status, reports on the growth of children exposed to ZIKV during pregnancy are still scarce.

This study describes the linear height growth velocity (GV) and nutritional status based on World Health Organization (WHO) standards of children exposed to ZIKV during pregnancy and cared for in a tertiary unit, which is a reference for tropical and infectious diseases in the Amazon. GV allows a better understanding of growth dynamics when compared to the isolated analysis of other anthropometric parameters, which are cumulatively affected by pre- and postnatal factors; moreover, adversities in maternal or fetal life may interfere with postnatal growth by producing a deceleration of GV [25,26,27,28,29].

## 2. Materials and Methods

Between March 2015 and August 2017, 77 women with laboratory-confirmed ZIKV infections during pregnancy and participants in the cohort of Redivo et al. [21] and Abtibol-Bernardino et al. [30] were invited to participate in the present study. Maternal ZIKV infection was confirmed in a blood or urine sample through real-time reverse transcriptase polymerase chain reaction (RT-PCR) detection. RT-PCR was performed following the protocol of Lanciotti et al. [31] at the Central Public Health Laboratory in Amazonas (LACEN-AM). Tests for Dengue virus and parvovirus B19 infections were performed by the Virology Laboratory at the Tropical Medicine Foundation Doutor Heitor Vieira Dourado (FMT-HVD), using Dengue Virus IgM Capture and Parvovirus B19 IgM kits (DxSelectTM Focus Diagnostics, Cypress, CA, USA). The detection of etiological agents of TORCH syndrome was mainly performed at the Clinical Analysis Laboratory of the FMT-HVD; however, new tests were not performed when the patient had recent test results from another (public or private network) laboratory. Malaria was investigated through a thick blood smear test in the FMT-HVD, but only in pregnant women with a positive clinical–epidemiological history.

Children born between March 2016 and June 2018 to mothers from the Redivo and Abtibol-Bernardino cohorts [21,30] were followed up at the outpatient clinic of the FMT-HVD, a tertiary reference center for tropical and infectious diseases in the state of Amazonas, for 42 months. At least two pediatric evaluations were performed during that follow-up period.

Children were monitored and evaluated for growth by a multidisciplinary team composed of a pediatrician, a pediatric nutritionist, a pediatric neurologist, an infectious disease specialist, a psychologist, a speech therapist, a physiotherapist, and an occupational therapist at the FMT-HVD outpatient clinic. Information on maternal history, prenatal history, and birth was obtained through interviews with the mothers, in the pregnant woman’s and child’s health booklet, and in the mothers’ electronic medical records. The pediatric clinical examination was performed by a pediatrician in accordance with the pediatric examination recommended by Nelson [32].

Anthropometric measurements of weight, height, head circumference, and GV were conducted according to international methods recommended by the WHO and the norms of the Brazilian Ministry of Health’s Manual for Monitoring Growth and Development, using a pediatric electronic scale and anthropometer [33,34,35,36,37]. Anthropometry was analyzed (i) at birth and (ii) during follow-up based, respectively, on charts internationally standardized by INTERGROWTH-21 [38,39] and the WHO (WHO Anthro software), the latter being an instrument used to monitor growth in the population of children aged 0 to 60 months [40]. For cases of prematurity, the chronological age was corrected during the first two years of life in accordance with Babson [41]; neuropsychomotor developmental disorders (NPMD) were defined according to Abtibol-Bernardino [30].

Growth velocity was defined by the difference between “B” and “A” heights divided by the time interval between the two assessments (minimum of 3 months and maximum of 12 months between them), being classified as (i) low growth (GV < 25th percentile) or (ii) adequate growth (GV ≥ 25th percentile). The GV was calculated in centimeters per year according to the Tanner methodology [42]. Maternal, birth, and neonatal variables were used to verify possible associations with GV.

Nutritional status was assessed using anthropometric indices: z-scores for body mass index for age (BMI/A), weight (W/A), and height (H/A). BMI/A is classified as (i) eutrophic (BMI/A ≤ +1 and ≥−2 standard deviation—SD), (ii) underweight (<−2 and ≥−3 SD), (iii) risk of overweight (≤+2 and >+1 SD), (iv) overweight (≤+3 and >+2 SD), or (v) obesity (>+3 SD), and these last three categories were unified and classified as inadequate BMI/A. Weight could be classified as adequate weight for age (W/A ≥ −2 and ≤+2 SD), low weight for age (W/A < −2 SD), or high weight for age (W/A > +2 SD). The z-score of head circumference for age at birth was used to classify children as microcephalic (HC/A < −2 SD), macrocephalic (>+2 SD), or normocephalic (between >+2 and <−2 SD). Linear height growth was assessed by height-for-age z-score (H/A) and classified as follows: (i) adequate height for age (H/A ≥ −2 and ≤+2 SD); (ii) low for age (≥−3 and <−2 SD) and (iii) very low for age (<−3 SD), unified and classified into inadequate H/A; and (iv) high for age (>+2 SD) [34,36,40].

This study is part of the project “Epidemiological, clinical, nutritional, virological, histopathological and immunological characteristics of ZIKV infection in pregnant women with acute exanthematous disease and its relationship with microcephaly or possible adverse outcomes in Manaus, Amazonas” approved by the Committee of Ethics in FMT-HVD (CAAE: 60168216.2.0000.0005, with approval number: 10806.030). Parents or legal guardians signed an informed consent form agreeing to the child’s participation.

Descriptive statistics were used representing categorical variables in frequency and percentage, and continuous variables were presented as means and standard deviations. We evaluated possible associations between categorical variables using the chi-square test, Student t-test, and Wilcoxon rank sum test for continuous variables. Statistical significance was considered when *p* < 0.05. Statistical analyses were performed using the R software, version 4.1.0 [43].

## 3. Results

Of the 77 children in the Abtibol-Bernardino [30] cohort, 71 were followed up regarding their anthropometric measurements and growth velocity. Of that group, 6 children were excluded because they attended a single assessment or went more than 12 months between assessments. The more detailed clinical characteristics of the individual children and their mothers are shown in Supplementary Table S1.

The mean number of evaluations over the follow-up was 3.8 (SD ± 1.96). The mean age at the first assessment was 7.1 months (SD ± 6.10), and at the last one it was 21.1 months (SD ± 8.93), with the maximum age at the last assessment being 41.2 months. Seventeen (24.2%) children had inadequate GV (low growth velocity). Table 1 presents the sociodemographic and prenatal clinical characteristics of the 71 pregnant women, according to the children’s growth velocity. The average age of mothers was 28 years old, and most of them had 9–11 schooling years (37 mothers, 52%). Most ZIKV infections occurred in the second trimester (29 women, 41%) of pregnancy. In children with inadequate GV, the infection frequency was greatest in the first trimester of pregnancy (7 children, 41%).

Of the children with low growth velocity, 71% were female, 5.9% were small for their gestational age, 5.9% had neonatal epileptic seizures, and 47% had altered neurological development upon examination. Except for gender (*p*: 0.044), the other variables did not show statistically significant differences. Four children had congenital microcephaly and severe neurological impairment. The other 67 were non-microcephalic children (60 normocephalic and 7 macrocephalic); of these, 24.2% (16 children) had neurological alterations, and 28.8% (19 children) had altered neuropsychomotor development. The frequencies of low growth among microcephalic and non-microcephalic patients were 25% (1 of 4 children) and 23.9% (16 of 67 children), respectively (Table 2).

**Figure 1:**
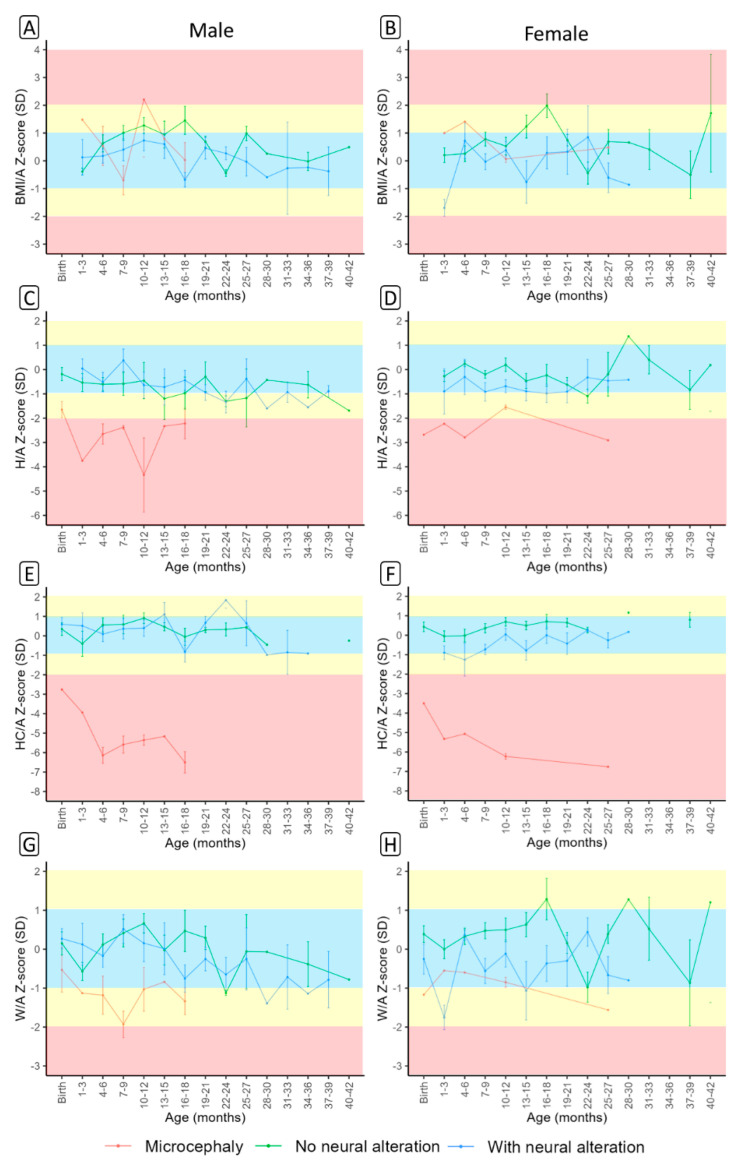
Body mass index (BMI/A) (**A**,**B**), height (H/A) (**C**,**D**), head circumference (HC/A) (**E**,**F**) and weight (W/A) (**G**,**H**) z-scores by sex between non-microcephalic (with and without neurological alteration) and microcephalic children according to age.

Based on gender, mean BMI/A, H/A, HC-A, and W/A z-scores were plotted against the presence of microcephaly and non-microcephaly (Figure 1). All scores varied over the follow-up period between boys and girls. Most children had normal BMI/A throughout the entire follow-up. However, two microcephalic patients presented BMI/A in the obesity range (Figure 1A,B). Microcephalic patients had low H/A and HC/A throughout the entire follow-up (Figure 1C–F), with a significant reduction in HC/A over the follow-up period (Figure 1E,F). Non-microcephalic subjects were within normal ranges for H/A, HC/A, and W/A, except for H/A scores for boys (Figure 1A–H). The anthropometric indices, at each assessment, of the 71 children during the pediatric follow-up for 42 months are presented in Supplementary Table S2.

In the final evaluation, this cohort had 48 (67.6%) eutrophic children, 1 (1.4%) with thinness, and 22 (31%) with inadequate BMI/A (risk of overweight/overweight and obesity). There were 66 (92.9%) with adequate W/A, 2 (2.8%) with low weight for their age, and 3 (4.2%) with high weight for their age. Furthermore, 64 (90%) had adequate height for their age, 6 (8.4%) had inadequate height for their age (short and very short), and one (1.4%) was tall for their age. At the last evaluation, the four children who were born with HC/A < −2 SD remained microcephalic; of the seven children who were born with HC/A > +2 SD, two remained macrocephalic, and the others remained normocephalic [34,36,40].

## 4. Discussion

The high incidence of ZIKV in the period of 2015–2016 in Brazil may have significantly affected GV in children exposed to ZIKV. In this study, we describe the GV alterations of children exposed in utero to ZIKV when both mothers and children were followed up in a cohort in the Brazilian Amazon [21,30]. We showed that 24.2% of children had inadequate GV, and the frequency of inadequate GV was similar between microcephalic and non-microcephalic patients (approximately 24%), indicating the need for microcephalic and non-microcephalic patients to be evaluated constantly and without predilections. Linear growth faltering in early childhood has long-term negative consequences such as cardiometabolic disease, obesity, diabetes, short stature in adolescence, and poor cognitive and school performance [44,45,46,47,48,49], and therefore children with altered GV must be constantly evaluated.

We evidenced similar aspects in relation to the anthropometric characteristics found by Aguiar et al. in a Brazilian cohort of women and children studied by the Oswaldo Cruz Foundation (FIOCRUZ) in Rio de Janeiro, Brazil [29]. However, we present GV for children with intrauterine exposure to ZIKV. GV allows the observation of a child’s linear growth compared to expected norms over a defined interval [50]. In addition, GV is more dynamic and valuable for assessing a child’s growth when compared to the size achieved or isolated anthropometric measurements [51,52,53,54]. Distinctly from Aguiar et al. [29], we found a lower frequency of growth alterations (24%) among microcephalic patients through a more reliable growth assessment tool. Additionally, we found growth alterations in non-microcephalic children, an aspect not evidenced by Aguiar et al. when assessing height. In a series of cases with 23 children exposed to the Zika virus who were born without microcephaly but who developed postnatal microcephaly and most of whom had neurological alterations, carried out in Pernambuco, Brazil, with children from the Pediatric Cohort of the Microcephaly Epidemic Research Group (MERG-PC), the monthly change in z-scores for length was –0.023 (95% CI −0.046 to 0.0001, *p* = 0.050), but their results for confidence intervals contain zero, so the data may indicate individual variations in opposite directions over time or that there was no change in the growth rate [55]. We showed statistically significant differences in GV between genders, with greater impairment of GV in females [33,56]; however, as distinctly described in the literature, boys, in general, tend to be more affected by height failures than girls in the first three to five years of life [29,34,44,57]. Ramos et al. showed no statistical difference between sexes in the monthly change in z-scores for length; however, for girls as a whole, there was decrease in z-score over time, but also with individual variations during the follow-up period [55]. Children with GV reduction, but who still have normal height, should be investigated early, even before their height becomes evidently compromised [28,33].

The frequency of inadequate GV in children with alterations in neurological exams was high (47%), although not statistically significant, when compared to children with adequate GV. In a cohort consisting of 29 children conducted at the Federal University of Rio de Janeiro (UFRJ) in Rio de Janeiro, Brazil, similar aspects were evidenced [9]. However, in that study the authors used anthropometric measurements (z-score for growth: weight, length, and HC) which are highly sensitive to short-term effects [58,59]. When we evaluated isolated anthropometric measures using scores corrected for age, we showed high variations over time, although higher rates of alteration were found in each age interval among microcephalic individuals, and trends were found in non-microcephalic individuals with neurological alteration. The greater the degree of neurological impairment, regardless of microcephaly at birth, the greater the impacts on growth, and such impacts can be associated with a greater degree of energy expenditure on sensory, behavioral, and cognitive factors related to secondary conditions (gastroesophageal reflux and constipation), which may lead to impaired food intake and absorption in children with neurological impairments [9,60,61].

In children with low growth rate, the infection frequently occurred in the first trimester of pregnancy (7 children, 41%). Other studies reveal an impairment of the growth pattern of children after birth in cases of congenital infection by other diseases such as HIV, syphilis and cytomegalovirus, for example, in the first trimester of pregnancy [62,63,64]. Different studies provide evidence of increased risks of adverse events (including miscarriage, fetal loss, low birth weight, and congenital malformations, especially in the central nervous system) in early ZIKV infections during pregnancy [13,58,59,65,66,67,68]. Neurological impairment seems to be greater in children whose mothers had an infection in the first trimester of pregnancy [30], and the probable risk of growth deficit seems to be associated with the presence of some degree of neurological impairment, even in children born without microcephaly [9]; nonetheless, nutritional and non-nutritional factors should be better evaluated, aiming to potentially understand the multifactorial etiology of impaired GV in children exposed to ZIKV. Small-for-gestational-age newborns following exposure to ZIKV during the first trimester of pregnancy were evidenced in another study [22]. Low birth weight was found in 2.8% of the children in the study, but 5.9% of those with low birth weight showed inadequate growth velocity. Such percentages are lower than those described in the national and international literature for children exposed to ZIKV [9,22,69,70,71,72].

This study has some limitations. The small sample size hinders a more robust statistical analysis. The lack of follow-up of a greater number of children born to mothers from Redivo’s cohort [21] is explained by the difficulty in keeping the children’s families adhering to continuous clinical evaluations, as many were born asymptomatic and without the CZVS phenotype. The number of children with microcephaly is small, and meaningful comparisons cannot be inferred, but we present findings that corroborate data from the literature, and we point out the need to assess both neurological and growth impairment in children exposed to ZIKV. There is no control group that allows assessing regional issues that may also be influencing children’s growth. In addition, it is necessary to evaluate other nutritional and non-nutritional factors (such as genetic and environmental factors) that can predict lower GV.

## 5. Conclusions

This study shows inadequate growth with low growth velocity in children with and without microcephaly, highlighting the need for continuous and thorough evaluation of all children born to mothers exposed to ZIKV during pregnancy in pediatric and multidisciplinary follow-up, but more studies are needed to support data capable of guiding clinical practice in the care of these children.

## Figures and Tables

**Table 1:** Sociodemographic and clinical characteristics of pregnant women with positive RT-PCR for Zika virus, distributed according to the children’s growth velocity, Amazonas, Brazil.

Characteristics	Low Growth Velocity n = 17 ^1^	Adequate Growth Velocity n = 54 ^1^	Overall n = 71 ^1^	*p*-Value ^2^
**Age, in years, mean (SD)**	30.0 (5.6)	27.5 (6.4)	28.0 (6.2)	0.25
**Years of schooling**				0.62
1–4 years	1/17 (5.9%)	4/54 (7.4%)	5/71 (7.0%)	
5–8 years	3/17 (18%)	5/54 (9.3%)	8/71 (11%)	
9–11 years	7/17 (41%)	30/54 (56%)	37/71 (52%)	
≥12 years	6/17 (35%)	15/54 (28%)	21/71 (30%)	
**Prenatal consultation**				>0.99
≥6 consultations	15/17 (88%)	48/54 (89%)	63/71 (89%)	
<6 consultations	2/17 (12%)	6/54 (11%)	8/71 (11%)	
**Trimester of ZIKV infection**				0.12
1st trimester	7/17 (41%)	10/54 (19%)	17/71 (24%)	
2nd trimester	4/17 (24%)	25/54 (46%)	29/71 (41%)	
3rd trimester	6/17 (35%)	19/54 (35%)	25/71 (35%)	
**Tobacco intake**	0/17 (0%)	1/54 (1.9%)	1/71 (1.4%)	>0.99
**Alcohol intake**	0/17 (0%)	2/54 (3.7%)	2/71 (2.8%)	>0.99
**Illicit drugs intake**	0/17 (0%)	0/54 (0%)	0/71 (0%)	>0.99
**Hypertensive disease**	4/17 (24%)	7/54 (13%)	11/71 (15%)	0.44
**Gestational diabetes**	0/17 (0%)	3/54 (5.6%)	3/71 (4.2%)	>0.99
**Intrauterine growth restriction**	1/17 (5.9%)	0/54 (0%)	1/71 (1.4%)	0.24
Coinfection occurrence *	2/17 (12%)	14/54 (26%)	16/71 (23%)	0.32
Herpes simplex type 1 and 2	2/17 (12%)	3/54 (5.6%)	5/71 (7.0%)	0.59
Parvovirus B19	1/17 (5.9%)	1/54 (1.9%)	2/71 (2.8%)	0.42
HIV	0/17 (0%)	2/54 (3.7%)	2/71 (2.8%)	>0.99
Epstein–Barr	0/17 (0%)	1/54 (1.9%)	1/71 (1.4%)	>0.99
Hepatitis B	0/17 (0%)	1/54 (1.9%)	1/71 (1.4%)	>0.99
Dengue	0/17 (0%)	4/54 (7.4%)	4/71 (5.6%)	0.57
Toxoplasmosis	0/17 (0%)	2/54 (3.7%)	2/71 (2.8%)	>0.99
Malaria	0/17 (0%)	1/54 (1.9%)	1/71 (1.4%)	>0.99
**Urinary tract infection**	3/17 (18%)	11/54 (20%)	14/71 (20%)	>0.99

^1^ n/N (%); Mean (SD); ^2^ Pearson’s chi-squared test; Fisher’s exact test; Wilcoxon rank sum test; * Some pregnant women had been coinfected with more than one infectious agent.

**Table 2:** Birth, neonatal, and postnatal clinical characteristics of children exposed in utero to the Zika virus, distributed according to the children’s growth velocity, Amazonas, Brazil.

Characteristics	Low Growth Velocity n = 17 ^1^	Adequate Growth Velocity n = 54 ^1^	Overall n = 71 ^1^	*p*-Value ^2^
**Gender**				0.044
Male	5/17 (29%)	31/54 (57%)	36/71 (51%)	
Female	12/17 (71%)	23/54 (43%)	35/71 (49%)	
**Childbirth Type**				0.85
Vaginal	8/17 (47%)	24/54 (44%)	32/71 (45%)	
Cesarean	9/17 (53%)	30/54 (56%)	39/71 (55%)	
**Apgar at 5th min <7**	0/16 (0%)	1/53 (1.9%)	1/69 (1.4%)	>0.99
**Gestational age, median (SD)**	39.00 (0.79)	39.00 (2.27)	39.00 (2.02)	0.88
**Prematurity**	0/17 (0%)	5/54 (9.3%)	5/71 (7.0%)	0.33
**Low birth weight (<2500 g)**	1/17 (5.9%)	4/54 (7.4%)	5/71 (7.0%)	>0.99
**Weight for gestational age at birth**				0.51
Appropriate for gestational age	16/17 (94%)	50/54 (93%)	66/71 (93%)	
Small for gestational age	1/17 (5.9%)	1/54 (1.9%)	2/71 (2.8%)	
Large for gestational age	0/17 (0%)	3/54 (5.6%)	3/71 (4.2%)	
**Length at birth**				0.67
Appropriate length at birth	16/17 (94%)	45/51 (88%)	61/68 (90%)	
Low length at birth	1/17 (5.9%)	6/51 (12%)	7/68 (10%)	
**Head circumference at birth**				>0.99
Normocephaly	15/17 (88%)	44/53 (83%)	60/70 (84%)	
Microcephaly	1/17 (5.9%)	3/53 (5.7%)	4/70 (5.7%)	
Macrocephaly	1/17 (5.9%)	6/53 (11%)	7/70 (10%)	
**Time Breastfeeding**				0.9
6 months	10/17 (59%)	28/54 (52%)	38/71 (54%)	
<6 months	7/17 (41%)	23/54 (43%)	30/71 (42%)	
Not breastfed	0/17 (0%)	3/54 (5.6%)	3/71 (4.2%)	
**Neonatal Complication**	3/17 (18%)	19/54 (35%)	22/71 (31%)	0.17
Neonatal Hyperbilirubinemia	1/17 (5.9%)	16/54 (30%)	17/71 (24%)	0.054
Intracranial hemorrhage				>0.99
Grades 1 and 2	0/17 (0%)	3/54 (5.6%)	3/71 (4.2%)	
Grades 3 and 4	0/17 (0%)	1/54 (1.9%)	1/71 (1.4%)	>0.99
Neonatal Sepsis	1/17 (5.9%)	4/54 (7.4%)	5/71 (7.0%)	>0.99
Hyaline Membrane Disease	0/17 (0%)	1/54 (1.9%)	1/71 (1.4%)	>0.99
Bronchopulmonary Dysplasia	0/17 (0%)	1/54 (1.9%)	1/71 (1.4%)	>0.99
Neonatal Epileptic Seizures	1/17 (5.9%)	2/54 (3.7%)	3/71 (4.2%)	0.57
**Dysphagia**	1/17 (5.9%)	6/54 (11%)	7/71 (9.9%)	>0.99
**Neurological Examination altered**	8/17 (47%)	15/54 (28%)	23/71 (32%)	0.14
NPMD * altered	6/17 (35%)	19/54 (35%)	25/71 (35%)	>0.99

^1^ n/N (%); mean (SD); ^2^ Pearson’s chi-squared test; Fisher’s exact test; Wilcoxon rank sum test. NPMD *: neuropsychomotor development.

## Data Availability

Not applicable.

## Supplementary Materials

The following supporting information can be downloaded at: https://www.mdpi.com/article/10.3390/v15030662/s1, Table S1: Sociodemographic and clinical characteristics, in absolute data, of pregnant women with positive RT-PCR and birth, neonatal, and postnatal clinical characteristics for Amazonas, Brazil. Table S2: Evolution of anthropometric indices of a cohort of 71 children exposed to the Zika virus during pregnancy, during pediatric follow-up for 42 months, Amazonas, Brazil.

## References

[1] Vue D., Tang Q. (2021). Zika Virus Overview: Transmission, Origin, Pathogenesis, Animal Model and Diagnosis. Zoonoses.

[2] WHO Zika Virus, Microcephaly and Guillain-Barré Syndrome Situation Report, 10 March 2016-World | ReliefWeb. https://reliefweb.int/report/world/who-zika-virus-microcephaly-and-guillain-barr-syndrome-situation-report-10-march-2016.

[3] Besnard M., Lastère S., Teissier A., Cao-Lormeau V.M., Musso D. (2014). Evidence of Perinatal Transmission of Zika Virus, French Polynesia, December 2013 and February 2014. Eurosurveillance.

[4] Foy B.D., Kobylinski K.C., Foy J.L.C., Blitvich B.J., da Rosa A.T., Haddow A.D., Lanciotti R.S., Tesh R.B. (2011). Probable Non-Vector-Borne Transmission of Zika Virus, Colorado, USA. Emerg. Infect. Dis..

[5] Musso D., Roche C., Robin E., Nhan T., Teissier A., Cao-Lormeau V.M. (2015). Potential Sexual Transmission of Zika Virus. Emerg. Infect. Dis..

[6] Magnus M.M., Espósito D.L.A., da Costa V.A., de Melo P.S., Costa-Lima C., da Fonseca B.A.L., Addas-Carvalho M. (2018). Risk of Zika Virus Transmission by Blood Donations in Brazil. Hematol. Transfus. Cell Ther..

[7] Mitsikas D., Gabrani C., Giannakou K., Lamnisos D. (2021). Intrauterine Exposure to Zika Virus and Hearing Loss within the First Few Years of Life: A Systematic Literature Review. Int. J. Pediatr. Otorhinolaryngol..

[8] Marbán-Castro E., Goncé A., Fumadó V., Romero-Acevedo L., Bardají A. (2021). Zika Virus Infection in Pregnant Women and Their Children: A Review. Eur. J. Obstet. Gynecol. Reprod. Biol..

[9] Prata-Barbosa A., Martins M.M., Guastavino A.B., da Cunha A.J.L.A. (2019). Effects of Zika Infection on Growth. J. Pediatr..

[10] Carvalho-Sauer R., da Costa M.C.N., Barreto F.R., Teixeira M.G. (2019). Congenital Zika Syndrome: Prevalence of Low Birth Weight and Associated Factors. Bahia, 2015–2017. Int. J. Infect. Dis..

[11] Aguilar Ticona J.P., Nery N., Ladines-Lim J.B., Gambrah C., Sacramento G., de Paula Freitas B., Bouzon J., Oliveira-Filho J., Borja A., Adhikarla H. (2021). Developmental Outcomes in Children Exposed to Zika Virus in Utero from a Brazilian Urban Slum Cohort Study. PLoS Negl. Trop. Dis..

[12] Santos G.P.G., de Gouveia M.T.O., Costa R.M.P.G., dos Santos A.M.R., Avelino F.V.S.D. (2020). Effects in the Development of Children Exposed to Zika Virus in the Fetal Period: An Integrative Review. Rev. Bras. Enferm..

[13] Maia A.M.P.C., Azevedo C.D.S.L., de Oliveira R.D.M.A.B., Barreto F.K.A., Rodrigues A.S.R., Simião A.R., Gomes I.P., Ribeiro E.M., Cavalcanti L.P.D.G. (2021). Neurological Growth and Development of Children Asymptomatic at Birth Whose Mothers Had Zika during Pregnancy. Rev. Soc. Bras. Med. Trop..

[14] Freitas D.A., Souza-Santos R., Carvalho L.M.A., Barros W.B., Neves L.M., Brasil P., Wakimoto M.D. (2020). Congenital Zika Syndrome: A Systematic Review. PLoS ONE.

[15] Sarno M., Sacramento G.A., Khouri R., do Rosário M.S., Costa F., Archanjo G., Santos L.A., Nery N., Vasilakis N., Ko A.I. (2016). Zika Virus Infection and Stillbirths: A Case of Hydrops Fetalis, Hydranencephaly and Fetal Demise. PLoS Negl. Trop. Dis..

[16] Auriti C., de Rose D.U., Santisi A., Martini L., Piersigilli F., Bersani I., Ronchetti M.P., Caforio L. (2021). Pregnancy and Viral Infections: Mechanisms of Fetal Damage, Diagnosis and Prevention of Neonatal Adverse Outcomes from Cytomegalovirus to SARS-CoV-2 and Zika Virus. Biochim. Biophys. Acta BBA -Mol. Basis Dis..

[17] Szaba F.M., Tighe M., Kummer L.W., Lanzer K.G., Ward J.M., Lanthier P., Kim I.J., Kuki A., Blackman M.A., Thomas S.J. (2018). Zika Virus Infection in Immunocompetent Pregnant Mice Causes Fetal Damage and Placental Pathology in the Absence of Fetal Infection. PLoS Pathog..

[18] Zhao Z., Li Q., Ashraf U., Yang M., Zhu W., Gu J., Chen Z., Gu C., Si Y., Cao S. (2022). Zika Virus Causes Placental Pyroptosis and Associated Adverse Fetal Outcomes by Activating GSDME. eLife.

[19] de Noronha L., Zanluca C., Azevedo M.L.V., Luz K.G., dos Santos C.N.D. (2016). Zika Virus Damages the Human Placental Barrier and Presents Marked Fetal. Mem. Inst. Oswaldo Cruz.

[20] Santos G.R., Pinto C.A.L., Prudente R.C.S., Bevilacqua E.M.A.F., Witkin S.S., Passos S.D. (2020). Histopathologic Changes in Placental Tissue Associated with Vertical Transmission of Zika Virus. Int. J. Gynecol. Pathol..

[21] de Redivo E.F., Menezes C.B., da Castilho M.C., Brock M., da Magno E.S., Saraiva M.D.G.G., Fernandes S.S.A., de Andrade A.B.C.A., Alecrim M.D.G.C., Martinez-Espinosa F.E. (2020). Zika Virus Infection in a Cohort of Pregnant Women with Exanthematic Disease in Manaus, Brazilian Amazon. Viruses.

[22] Souza J.P., Méio M.D.B.B., de Andrade L.M., Figueiredo M.R., Gomes Junior S.C., Pereira Junior J.P., Brickley E., Moreira M.E.L. (2021). Adverse Fetal and Neonatal Outcomes in Pregnancies with Confirmed Zika Virus Infection in Rio de Janeiro, Brazil: A Cohort Study. PLoS Negl. Trop. Dis..

[23] dos Santos F.A.A., Magno L.D., Araújo B.C.L., Taguchi C.K., Gurgel R.Q. (2021). Evaluation of Food Function in Children Microcephaly by Zika Virus: Two-Year Follow-Up. Res. Soc. Dev..

[24] Soares F., Abranches A.D., Villela L., Lara S., Araújo D., Nehab S., Silva L., Amaral Y., Clair Junior S.G., Pone S. (2019). Zika Virus Infection in Pregnancy and Infant Growth, Body Composition in the First Three Months of Life: A Cohort Study. Nat. Rep..

[25] Hosseini S.M., Maracy M.R., Sarrafzade S., Kelishadi R. (2014). Child Weight Growth Trajectory and Its Determinants in a Sample of Iranian Children from Birth until 2 Years of Age. Int. J. Prev. Med..

[26] de Fonseca P.C.A., de Carvalho C.A., Ribeiro S.A.V., Nobre L.N., Pessoa M.C., Ribeiro A.Q., Priore S.E., do Franceschini S.C.C. (2017). Determinants of the Mean Growth Rate of Children under the Age of Six Months: A Cohort Study. Cienc. Saude Coletiva.

[27] Regnault N., Botton J., Forhan A., Hankard R., Thiebaugeorges O., Hillier T.A., Kaminski M., Heude B., Charles M.A. (2010). Determinants of Early Ponderal and Statural Growth in Full-Term Infants in the EDEN Mother-Child Cohort Study. Am. J. Clin. Nutr..

[28] Sbp Departamento de Nutrologia (2021). Manual de Orientação de Avaliação Nutricional da Criança e do Adolescente.

[29] de Aguiar E.B., Pone S.M., Junior S.C.D.S.G., Soares F.V.M., Zin A.A., Vasconcelos Z.F.M., Ribeiro C.T.M., Junior J.P.P., Moreira M.E.L., Nielsen-Saines K. (2022). Anthropometric Parameters of Children with Congenital Zika Virus Exposure in the First Three Years of Life. Viruses.

[30] Abtibol-Bernardino M.R., Peixoto L.d.F.A.d.A., da Castilho M.C., Bôtto-Menezes C.H.A., Benzecry S.G., Otani R.H., Rodrigues G.R.I., Chaves B.C.S., de Oliveira G.A., de Rodrigues C.S. (2022). Would Zika Virus Infection in Pregnancy Be a Sentence of Poor Neurological Prognosis for Exposed Children? Neurodevelopmental Outcomes in a Cohort from Brazilian Amazon. Viruses.

[31] Lanciotti R.S., Kosoy O.L., Laven J.J., Velez J.O., Lambert A.J., Johnson A.J., Stanfield S.M., Duffy M.R. (2008). Genetic and Serologic Properties of Zika Virus Associated with an Epidemic, Yap State, Micronesia, 2007. Emerg. Infect. Dis..

[32] Kliegman R.M., Behrman R.E., Jenson H.B., Stanton B.F. (2009). Nelson Tratado de Pediatria.

[33] World Health Organization (2009). Nutrition for Health and Development. WHO Child Growth Standards: Growth Velocity Based on Weight, Length and Head Circumference: Methods and Development.

[34] WHO World Health Organization (2006). WHO Child Growth Standard-Length/Height-for-Age, Weight-for-Age, Weight-for-Length, Weight-for-Height and Body Mass Index-for-Age Methods and Development.

[35] WHO (1995). Physical Status: The Use of and Interpretation of Anthropometry, Report of a WHO Expert Committee.

[36] da Saúde Brasil M. (2011). Orientações Coleta Análise Dados Antropométricos Em Serviços de Saúde.

[37] da Saúde Brasil M., de Atenção à Saúde S., Brasil (2012). Atenção Básica Cadernos de Biblioteca Virtual em Saúde do Ministério da Saúde. Saúde da Criança: Crescimento e Desenvolvimento.

[38] Villar J., Ismail L.C., Victora C.G., Ohuma E.O., Bertino E., Altman D.G., Lambert A., Papageorghiou A.T., Carvalho M., Jaffer Y.A. (2014). International Standards for Newborn Weight, Length, and Head Circumference by Gestational Age and Sex: The Newborn Cross-Sectional Study of the INTERGROWTH-21st Project. Lancet.

[39] University of Oxford, INTERGROWTH-21 (2009). INTERGROWTH-21-The International Fetal and Newborn Growth Standards for the 21st Century.

[40] WHO WHO Anthro Software. Department of Nutrition: Geneva, Switzerland..

[41] Babson S.G. (1970). Growth of Low-Birth-Weight Infants. J. Pediatr..

[42] Tanner J.M., Whitehouse R.H. (1976). Clinical Longitudinal Standards for Height, Weight, Height Velocity, Weight Velocity, and Stages of Puberty. Arch. Dis. Child.

[43] R Core Team (2020). R: A Language and Environment for Statistical Computing.

[44] Calu Costa J., Blumenberg C., Victora C. (2021). Growth Patterns by Sex and Age among Under-5 Children from 87 Low-Income and Middle-Income Countries. BMJ Glob. Health.

[45] Grantham-McGregor S., Cheung Y.B., Cueto S., Glewwe P., Richter L., Strupp B. (2007). Developmental Potential in the First 5 Years for Children in Developing Countries. Lancet.

[46] Adair L.S., Fall C.H.D., Osmond C., Stein A.D., Martorell R., Ramirez-Zea M., Sachdev H.S., Dahly D.L., Bas I., Norris S.A. (2013). Associations of Linear Growth and Relative Weight Gain during Early Life with Adult Health and Human Capital in Countries of Low and Middle Income: Findings from Five Birth Cohort Studies. Lancet.

[47] Prado E.L., Yakes Jimenez E., Vosti S., Stewart R., Stewart C.P., Somé J., Pulakka A., Ouédraogo J.B., Okronipa H., Ocansey E. (2019). Path Analyses of Risk Factors for Linear Growth Faltering in Four Prospective Cohorts of Young Children in Ghana, Malawi and Burkina Faso. BMJ Glob. Health.

[48] Sterling R., Miranda J.J., Gilman R.H., Cabrera L., Sterling C.R., Bern C., Checkley W. (2012). Early Anthropometric Indices Predict Short Stature and Overweight Status in a Cohort of Peruvians in Early Adolescence. Am. J. Phys. Anthropol..

[49] Rolland-Cachera M.F. (2005). Rate of Growth in Early Life: A Predictor of Later Health?. Adv. Exp. Med. Biol..

[50] Samson-Fang L., Stevenson R.D. (1998). Linear Growth Velocity in Children with Cerebral Palsy. Dev. Med. Child Neurol..

[51] Ghaemmaghami P., Ayatollahi S.M.T., Alinejad V., Haem E. (2015). Longitudinal Standards for Growth Velocity of Infants from Birth to 4 Years Born in West Azerbaijan Province of Northwest Iran. Epidemiol. Health.

[52] de Onis M., Siyam A., Borghi E., Onyango A.W., Piwoz E., Garza C. (2011). Comparison of the World Health Organization Growth Velocity Standards With Existing US Reference Data. Pediatrics.

[53] Ayatollahi S.M.T. (2004). Infants Body Mass Index Reference Curves for Iran. J. Res. Med. Sci..

[54] De A.M., Costello L. (1989). Growth Velocity and Stunting in Rural Nepal. Arch. Dis. Child.

[55] Ramos R.C.F., de Barros Miranda-Filho D., Martelli C.M.T., de Araújo T.V.B., Wanderley Rocha M.A., van der Linden V., de Carvalho M.D.C.G., Rodrigues L.C., Montarroyos U.R., de Souza W.V. (2022). Characteristics of Children of the Microcephaly Epidemic Research Group Pediatric Cohort Who Developed Postnatal Microcephaly. Sci. Rep..

[56] Kattula D., Sarkar R., Sivarathinaswamy P., Velusamy V., Venugopal S., Naumova E.N., Muliyil J., Ward H., Kang G. (2014). The First 1000 Days of Life: Prenatal and Postnatal Risk Factors for Morbidity and Growth in a Birth Cohort in Southern India. BMJ Open.

[57] Wamani H., Åstrøm A.N., Peterson S., Tumwine J.K., Tylleskär T. (2007). Boys Are More Stunted than Girls in Sub-Saharan Africa: A Meta-Analysis of 16 Demographic and Health Surveys. BMC Pediatr..

[58] Hollanders J.J., van der Pal S.M., van Dommelen P., Rotteveel J., Finken M.J.J. (2017). Growth Pattern and Final Height of Very Preterm vs. Very Low Birth Weight Infants. Pediatr. Res..

[59] Simon L., Nusinovici S., Flamant C., Cariou B., Valérie R., Gascoin G., Darmaun D., Jean-Christophe R., Hanf M. (2017). Post-Term Growth and Cognitive Development at 5 Years of Age in Preterm Children: Evidence from a Prospective Population-Based Cohort. PLoS ONE.

[60] Quitadamo P., Thapar N., Staiano A., Borrelli O. (2016). Gastrointestinal and Nutritional Problems in Neurologically Impaired Children. Eur. J. Paediatr. Neurol..

[61] Sullivan P., Lambert B., Rose M., Ford-Adams M., Johnson A., Griffiths P. (2000). Prevalence and Severity of Feeding and Nutritional Problems in Children with Neurological Impairment: Oxford Feeding Study. Dev. Med. Child Neurol..

[62] Tagarro A., del Valle R., Dominguez-Rodríguez S., Baquero-Artigao F., Noguera-Julian A., Vives-Onõs I., Santos M., Hawkins M.M., Pérez-Seoane B., Medina G. (2019). Growth Patterns in Children with Congenital Cytomegalovirus Infection. Pediatr. Infect. Dis. J..

[63] Shen H., Sun  M., Liu A. (2009). Clinical Features of 121 Infants below 3 Months of Age with Congenital Syphilis-PubMed. Zhonghua Er Ke Za Zhi.

[64] Desmonde S., Goetghebuer T., Thorne C., Leroy V. (2016). Health and Survival of HIV Perinatally Exposed but Uninfected Children Born to HIV-Infected Mothers. Curr. Opin. HIV AIDS.

[65] Brasil P., Pereira J.P., Moreira M.E., Ribeiro Nogueira R.M., Damasceno L., Wakimoto M., Rabello R.S., Valderramos S.G., Halai U.-A., Salles T.S. (2016). Zika Virus Infection in Pregnant Women in Rio de Janeiro. N. Engl. J. Med..

[66] van der Linden V., Pessoa A., Dobyns W., James Barkovich A., van der Linden H., Rolim Filho E.L., Ribeiro E.M., de Carvalho Leal M., de Araújo Coimbra P.P., de Fátima Viana Vasco Aragão M. (2016). Description of 13 Infants Born during October 2015-January 2016 with Congenital Zika Virus Infection without Microcephaly at Birth - Brazil. Morb. Mortal. Wkly. Rep..

[67] Cardona-Ospina J.A., Zapata M.F., Grajales M., Arias M.A., Grajales J., Bedoya-Rendón H.D., González-Moreno G.M., Lagos-Grisales G.J., Suárez J.A., Rodríguez-Morales A.J. (2021). Physical Growth and Neurodevelopment of a Cohort of Children after 3.5 Years of Follow-up from Mothers with Zika Infection during Pregnancy-Third Report of the ZIKERNCOL Study. J. Trop. Pediatr..

[68] Schirmer D.A., Kawwass J.F. (2016). Epidemiology, Virology, and Pathogenesis of the Zika Virus: From Neglected Tropical Disease to a Focal Point of International Attention. Semin. Reprod. Med..

[69] Cooper H.J., Iwamoto M., Lash M., Conners E.E., Paladini M., Slavinski S., Fine A.D., Kennedy J., Heinke D., Ciaranello A. (2019). Maternal Zika Virus Infection: Association With Small-for-Gestational-Age Neonates and Preterm Birth. Obstet. Gynecol..

[70] Walker C.L., Merriam A.A., Ohuma E.O., Dighe M.K., Gale M., Rajagopal L., Papageorghiou A.T., Gyamfi-Bannerman C., Adams Waldorf K.M. (2018). Femur-Sparing Pattern of Abnormal Fetal Growth in Pregnant Women from New York City After Maternal Zika Virus Infection. Am. J. Obstet. Gynecol..

[71] Lee A.C.C., Katz J., Blencowe H., Cousens S., Kozuki N., Vogel J.P., Adair L., Baqui A.H., Bhutta Z.A., Caulfield L.E. (2013). National and Regional Estimates of Term and Preterm Babies Born Small for Gestational Age in 138 Low-Income and Middle-Income Countries in 2010. Lancet Glob. Health.

[72] Hoen B., Schaub B., Funk A.L., Ardillon V., Boullard M., Cabié A., Callier C., Carles G., Cassadou S., Césaire R. (2018). Pregnancy Outcomes after ZIKV Infection in French Territories in the Americas. N. Engl. J. Med..

