# Supplementary material for: Growth Velocity and Nutritional Status in Children Exposed to Zika Virus during Pregnancy from Amazonas Cohort, Brazil
Source: Viruses. 2023 Mar 1;15(3):662. doi: 10.3390/v15030662 (PMC10056230; doi:10.3390/v15030662)
Supplement: Supplementary file 1 [file viruses-15-00662-s001.zip › viruses-2168740-supplementary.pdf]

Table Supplementary 1. Sociodemographic and clinical characteristics, in absolute data, of pregnant women with positive RT-PCR and birth, neonatal, and postnatal clinical characteristics for Amazonas, Brazil.

Case	Maternal characteristics								Children's characteristics											
	Age	Year of schooling	Number of prenatal consultation	Trimester of ZIKV infection	Tobacco or/and alcohol or/and Illicit drugs intake	Disease Gestational	Coinfection occurrence	Urinary tract infection	Gender	Type of delivery	Apgar at 5th min<7	Gestational age	Pre-maturity	Birth weight (gram)	Length at birth (cm)	HC at birth (cm)	Breast-feeding (months)	Neonatal complication	Dysphagia	Neurological or NPMD dhanged
1	30	9-11	13	2nd	No	HD	No	No	Female	Cesarea	No	40.0	No	3120	47	35	6 mo	No	No	Neu and NPMD
2	23	≥12	5	1st	No	No	HP, B19	No	Male	Vaginal	-	40.0	No	3755	49	37	<6 mo	No	No	NPMD
3	27	≥12	5	3rd	No	No	No	No	Female	Cesarea	No	39.0	No	3500	50	35	6 mo	No	No	Neu and NPMD
5	30	≥12	6	2nd	No	HD	No	No	Male	Vaginal	No	39.0	No	2810	48	34	6 mo	No	No	NPMD
6	30	≥12	6	3rd	No	No	No	No	Male	Cesarea	No	36.0	Yes	2940	-	34	<6 mo	No	No	NPMD
8	18	9-11	5	2nd	No	No	No	No	Female	Vaginal	No	33.1	Yes	2145	44	31.4	6 mo	NHB, IHG and NS	No	Neu and NPMD
12*	22	1-4	7	1st	No	No	No	No	Female	Vaginal	No	39.1	No	2710	44	29	<6 mo	No	Yes	Neu and NPMD
19	40	≥12	10	1st	No	No	T	No	Male	Vaginal	No	39.0	No	3650	49	34	<6 mo	IHG	No	Neu and NPMD
22*	23	5-8	7	1st	No	HD	No	No	Male	Cesarea	No	40.0	No	3600	48	31	<6 mo	NEC	Yes	Neu and NPMD
24	20	1-4	7	2nd	No	GD	No	Yes	Male	Cesarea	No	39.0	No	4256	51	37	<6 mo	No	No	Neu and NPMD
25*	22	≥12	13	1st	No	No	No	No	Male	Cesarea	No	38.0	No	2516	44,5	30	No	NHB and NEC	Yes	Neu and NPMD
31	22	5-8	6	2nd	No	No	HIV	Yes	Male	Cesarea	No	39.0	No	3440	49,5	36	No	NHB	No	Neu and NPMD
33	36	≥12	7	2nd	No	No	No	No	Male	Cesarea	No	39.0	No	3590	50	36	<6 mo	No	No	Neu and NPMD
35	26	9-11	9	1st	No	No	No	Yes	Male	Cesarea	No	40.0	No	3064	50	33	<6 mo	IHG	Yes	Neu and NPMD

40	19	9-11	7	2nd	Yes	No	No	No	Female	Cesarea	No	39.0	No	2232	45	32	6 mo	No	No	NPMD
42	38	≥12	8	2nd	No	No	No	No	Female	Cesarea	No	39.0	No	3600	48	34	6 mo	No	No	Neu and NPMD
49	25	9-11	6	3rd	No	HD	No	No	Male	Cesarea	No	34.5	Yes	2110	41	32	<6 mo	NHB and NS	Yes	NPMD
53	25	9-11	5	2nd	No	No	No	No	Female	Vaginal	Yes	26.0	Yes	770	-	-	6 mo	NHB, IHG, NS, HMD and BPD	Yes	Neu and NPMD
55	17	5-8	9	3rd	No	No	No	No	Male	Vaginal	No	39.0	No	3260	47	33	6 mo	No	No	Neu and NPMD
57	32	≥12	9	2nd	No	No	No	No	Female	Cesarea	No	38.0	No	3100	46	36	6 mo	No	No	Neu and NPMD
60	17	1-4	5	2nd	No	No	HB	No	Male	Vaginal	No	40.0	No	2725	47	32	<6 mo	No	No	DNPM
65*	23	9-11	10	1st	No	No	No	Yes	Male	Cesarea	No	38.0	No	2828	46	30	6 mo	IHG and NEC	Yes	Neu and NPMD
67	36	≥12	10	3rd	No	No	No	No	Female	Cesarea	No	39.0	No	2895	48	33	6 mo	NHB	No	NPMD
68	28	9-11	10	2nd	No	No	No	No	Male	Cesarea	No	39.0	No	3030	48	33	<6 mo	NHB	No	NPMD
71	27	9-11	10	2nd	No	No	HIV	No	Male	Cesarea	No	38.4	No	3725	52,5	37	No	No	No	Neu and NPMD

HC: head circumference; NPMD: neuropsychomotor development, Disease Gestational: HD: Hypertensive disease; GD: Gestational diabetes; IGR: Intrauterine growth restriction.

Coinfection occurrence: HP: Herpes simplex type 1 and 2; B19: Pavovirus B19; HIV; EB: Epstein-Barr; HB: Hepatitis B; D: Dengue; T: Toxoplasmose; M: Malaria.

Neonatal complication: NHB: Neonatal Hyperbilirrubinemia; IHG: Intracranial hemorrhage Grades 1 and 2 or Grades 3 and 4; NS: Neonatal sepsis; HMD: Hyaline Membrane Disease; BPD: Bronchopulmonary Dysplasia ; NEC: Neonatal Epleptic Crises.

*Microcephaly children.

Table Supplementary 2. Evolution of anthropometric indices of a cohort of 71 children exposed to the *Zika virus* during pregnancy, during pediatric follow-up for 42 months, Amazonas, Brazil.

Case	Gender	Z score Growth, and Age	Birth	Evaluation 1	Evaluation 2	Evaluation 3	Evaluation 4	Evaluation 5	Evaluation 6
		AE, in months,	-	6	8	10	12	24	26
		W/A-Z	-0,36	-0,20	-0,48	-0,60	-0,88	0,26	0,02
		H/A-Z	-1,38	-0,53	-0,35	-1,31	-0,32	-0,46	-0,38
		BMI/A-Z	-	0,13	-0,41	0,21	-0,99	0,73	0,32
1	Female	HC/A-Z	0,22	-0,61	0,20	-0,34	-0,07	0,07	0,03
		AE, in months,	-	0	1	5	20	-	-
		W/A-Z	0,88	0,97	1,03	0,42	-0,03	-	-
		H/A-Z	-0,57	0,83	0,97	0,27	0,05	-	-
		BMI/A-Z	-	0,78	0,72	0,35	-0,06	-	-
2	Male	HC/A-Z	2,21	2,49	1,30	1,82	0,67	-	-
		AE, in months,	-	6	9	11	16	18	22
		W/A-Z	0,90	0,07	0,54	0,90	0,46	0,46	0,81
		H/A-Z	0,85	-0,86	-0,67	-0,36	-1,67	-1,66	-1,45
		BMI/A-Z	-	0,74	1,24	1,51	1,97	1,98	2,31
3	Female	HC/A-Z	1,38	-0,99	0,76	0,83	0,82	0,53	0,38
		AE, in months,	-	1	2	5	-	-	-
		W/A-Z	-0,13	-0,39	0,34	0,12	-	-	-
		H/A-Z	-1,02	-0,82	0,27	-0,62	-	-	-
		BMI/A-Z	-	0,05	0,25	0,62	-	-	-
4	Female	HC/A-Z	-0,37	-0,08	0,71	0,30	-	-	-
		AE, in months,	-	10	11	26	29	-	-
		W/A-Z	-1,06	-1,05	-1,20	-1,63	-1,39	-	-
5	Male	H/A-Z	-0,77	-1,44	-1,99	-1,48	-1,60	-	-

		BMI/A-Z	-	-0,27	-0,01	-1,06	-0,59	-	-
		HC/A-Z	0,08	-1,34	-0,83	-1,11	-0,98	-	-
		AE, in months,	-	2	4	6	9	10	20
		W/A-Z	0,59	1,03	0,21	-0,25	0,41	0,06	0,34
		H/A-Z	-	-0,5	-0,59	-0,47	-0,92	-0,96	-0,72
		BMI/A-Z	-	1,9	0,75	0,03	1,25	0,83	1,11
6	Male	HC/A-Z	1,13	-1,65	-1,12	-0,04	0,25	-0,3	0,25
		AE, in months,	-	1	2	3	6	7	12
		W/A-Z	0,90	0,05	-0,66	0,74	1,29	1,53	1,71
		H/A-Z	-0,62	-0,32	-1,47	0,24	-0,19	-0,33	0,75
		BMI/A-Z	-	0,29	0,24	0,82	1,86	2,29	1,78
7	Female	HC/A-Z	1,65	-0,44	1,05	0,92	0,77	0,47	1,08
		AE, in months,	-	10	12	13	25	27	30
		W/A-Z	0,68	-0,43	-0,41	-0,38	0,81	-0,34	-0,8
		H/A-Z	0,24	-0,73	-0,19	-0,67	-0,59	0,12	-0,42
		BMI/A-Z	-	-0,04	-0,44	0,00	1,62	-0,68	-0,86
8	Female	HC/A-Z	0,62	0,22	0,36	0,31	0,26	0,37	0,17
		AE, in months,	-	1	3	4	6	9	11
		W/A-Z	-0,22	-0,21	-1,22	0,14	0,54	0,88	0,65
		H/A-Z	-1,35	-0,41	-1,66	-1,31	-1,70	-0,16	-1,09
		BMI/A-Z	-	0,00	-0,36	1,21	2,03	1,29	1,71
9	Male	HC/A-Z	-0,78	-0,27	-1,27	2,08	1,81	1,49	0,97
		AE, in months,	-	3	5	10	-	-	-
		W/A-Z	1,34	1,84	1,74	1,75	-	-	-
		H/A-Z	1,13	0,26	0,41	0,65	-	-	-
10	Female	BMI/A-Z	-	2,31	2,03	1,90	-	-	-

		HC/A-Z	1,94	0,76	0,36	0,85	-	-	-
		AE, in months,	-	1	2	6	11	22	40
		W/A-Z	0,01	-0,61	-0,98	-1,58	-1,54	-1,89	-1,37
		H/A-Z	-1,60	0,32	-0,55	-1,37	-1,39	-1,92	-1,72
		BMI/A-Z	-	-1,13	-0,94	-1,07	-1,02	-0,96	-0,41
11	Female	HC/A-Z	-0,78	-1,62	-0,62	-0,28	-0,21	0,03	-0,59
		AE, in months,	-	2	5	9	10	11	26
		W/A-Z	-1,17	-0,55	-0,6	-0,66	-0,8	-1,09	-1,56
		H/A-Z	-2,68	-2,23	-2,79	-1,37	-1,63	-1,65	-2,91
		BMI/A-Z	-	1	1,41	0,18	0,19	-0,18	0,48
12	Female	HC/A-Z	-3,50	-5,33	-5,07	-5,99	-6,22	-6,47	-6,76
		AE, in months,	-	11	13	18	21	-	-
		W/A-Z	-0,12	-0,39	-0,41	-0,07	0,03	-	-
		H/A-Z	-0,41	-0,75	-0,99	-0,82	-1,13	-	-
		BMI/A-Z	-	0,05	0,22	0,57	0,98	-	-
13	Female	HC/A-Z	0,52	-0,46	-0,31	-0,20	0,21	-	-
		AE, in months,	-	7	9	11	19	24	-
		W/A-Z	2,64	0,29	0,15	0,06	0,23	0,38	-
		H/A-Z	1,80	-0,55	-0,18	-0,08	0,15	-0,72	-
		BMI/A-Z	-	0,81	0,32	0,13	0,18	1,13	-
14	Female	HC/A-Z	0,49	-0,25	-0,45	0,42	0,44	0,6	-
		AE, in months,	-	1	3	6	8	13	19
		W/A-Z	0,98	0,87	0,94	1,17	1,34	1,20	0,90
		H/A-Z	-0,14	0,20	0,34	0,34	-0,12	0,96	0,44
		BMI/A-Z	-	1,06	1,02	1,30	1,89	0,92	0,90
15	Female	HC/A-Z	2,68	1,24	1,69	2,37	2,61	1,66	1,77

		AE, in months,	-	7	24	-	-	-	-
		W/A-Z	1,43	0,51	0,15	-	-	-	-
		H/A-Z	-0,17	0,96	-1,09	-	-	-	-
		BMI/A-Z	-	-0,05	1,13	-	-	-	-
16	Female	HC/A-Z	1,92	0,75	0,57	-	-	-	-
		AE, in months,	-	10	-	-	-	-	-
		W/A-Z	1,01	0,72	-	-	-	-	-
		H/A-Z	0,49	1,42	-	-	-	-	-
		BMI/A-Z	-	-0,10	-	-	-	-	-
17	Female	HC/A-Z	0,59	0,37	-	-	-	-	-
		AE, in months,	-	2	6	8	-	-	-
		W/A-Z	-0,04	-1,44	-1,61	-1,57	-	-	-
		H/A-Z	-0,41	0,03	-3,76	-1,56	-	-	-
		BMI/A-Z	-	-2,00	0,91	-0,90	-	-	-
18	Female	HC/A-Z	-0,78	-0,55	-0,57	-0,23	-	-	-
		AE, in months,	-	0	1	3	6	8	9
		W/A-Z	0,97	-0,05	-1,83	0,33	0,34	0,54	0,90
		H/A-Z	-0,17	1,07	-0,28	1,22	0,98	0,97	0,72
		BMI/A-Z	-	-0,85	-2,45	-0,49	-0,28	0,00	0,66
19	Male	HC/A-Z	0,08	-0,64	-0,61	-1,21	-1,51	-0,90	-0,10
		AE, in months,	-	11	-	-	-	-	-
		W/A-Z	0,02	1,32	-	-	-	-	-
		H/A-Z	-0,17	1,65	-	-	-	-	-
		BMI/A-Z	-	0,58	-	-	-	-	-
20	Male	HC/A-Z	0,08	1,87	-	-	-	-	-
		AE, in months,	-	3	6	10	12	14	18

		W/A-Z	-0,90	-2,16	0,00	-0,42	-0,89	-0,57	-0,46
		H/A-Z	-3,24	-2,85	-1,70	-3,60	-2,12	-2,38	-2,77
		BMI/A-Z	-	-0,70	1,34	2,33	0,51	1,18	1,74
21	Male	HC/A-Z	0,44	-1,44	1,36	0,90	1,34	1,02	1,19
		AE, in months,	-	1	3	5	9	14	17
		W/A-Z	0,50	-1,13	0,33	-0,41	-0,47	-0,84	-0,74
		H/A-Z	-1,18	-3,75	-2,04	-3,62	-5,86	-2,33	-2,53
		BMI/A-Z	-	1,48	2,09	2,37	4,27	0,79	1,14
22	Male	HC/A-Z	-2,70	-3,94	-5,32	-5,05	-5,62	-5,17	-6,26
		AE, in months,	-	4	16	-	-	-	-
		W/A-Z	0,25	0,18	-0,54	-	-	-	-
		H/A-Z	-0,05	-1,81	-0,83	-	-	-	-
		BMI/A-Z	-	1,68	-0,04	-	-	-	-
23	Male	HC/A-Z	1,24	-0,06	0,62	-	-	-	-
		AE, in months,	-	9	-	-	-	-	-
		W/A-Z	2,21	2,69	-	-	-	-	-
		H/A-Z	1,01	1,98	-	-	-	-	-
		BMI/A-Z	-	2,23	-	-	-	-	-
24	Male	HC/A-Z	2,46	2,24	-	-	-	-	-
		AE, in months,	-	3	6	15	-	-	-
		W/A-Z	-1,47	-1,96	-1,62	-1,34	-	-	-
		H/A-Z	-2,28	-2,7	-1,42	-1	-	-	-
		BMI/A-Z	-	-0,49	-1,07	-1,05	-	-	-
25	Male	HC/A-Z	-2,82	-7,13	-6,39	-7,55	-	-	-
		AE, in months,	-	1	10	32	41	-	-
26	Female	W/A-Z	1,34	0,83	1,89	2,95	3,78	-	-

		H/A-Z	0,49	0,79	1,57	2,15	2,09	-	-
		BMI/A-Z	-	0,60	1,43	2,56	3,84	-	-
		HC/A-Z	0,22	0,49	1,98	-	-	-	-
		AE, in months,	-	4	6	-	-	-	-
		W/A-Z	1,63	0,89	0,39	-	-	-	-
		H/A-Z	2,83	0,16	-0,66	-	-	-	-
		BMI/A-Z	-	1,06	1,02	-	-	-	-
27	Male	HC/A-Z	-0,70	0,84	0,30	-	-	-	-
		AE, in months,	-	1	3	4	6	7	27
		W/A-Z	-0,86	-1,07	-0,85	-0,87	-0,29	-0,55	-0,07
		H/A-Z	-1,74	-1,37	-0,73	-0,86	-1,35	-1,08	-0,43
		BMI/A-Z	-	-0,51	-0,61	-0,51	0,68	0,12	0,26
28	Male	HC/A-Z	-2,00	-1,92	-1,73	-0,65	-1,36	-0,05	-0,46
		AE, in months,	-	4	7	8	10	14	19
		W/A-Z	0,91	-0,15	-0,11	0,38	0,75	0,92	0,87
		H/A-Z	0,49	0,53	-0,89	-0,65	-0,34	0,15	-0,14
		BMI/A-Z	-	-0,61	0,53	1,01	1,28	1,15	1,32
29	Female	HC/A-Z	1,11	-2,30	0,05	0,27	0,86	0,68	0,70
		AE, in months,	-	4	7	12	14	17	29
		W/A-Z	0,38	1,41	1,13	1,46	1,59	1,57	1,28
		H/A-Z	-1,60	0,81	-0,19	-0,44	-0,06	-0,21	1,37
		BMI/A-Z	-	1,30	1,64	2,31	2,23	2,34	0,66
30	Female	HC/A-Z	0,52	1,06	0,09	0,42	0,67	0,56	1,16
		AE, in months,	-	6	-	-	-	-	-
		W/A-Z	0,48	1,84	-	-	-	-	-
31	Male	H/A-Z	0,12	-0,62	-	-	-	-	-

		BMI/A-Z	-	2,84	-	-	-	-	-
		HC/A-Z	1,72	1,17	-	-	-	-	-
		AE, in months,	-	2	4	6	12	15	18
		W/A-Z	-1,65	-0,66	-0,06	-0,10	0,18	0,02	0,46
		H/A-Z	-3,86	-1,74	-0,30	-0,86	-2,87	-1,59	-1,14
		BMI/A-Z	-	0,44	0,15	0,52	2,52	1,31	1,55
32	Female	HC/A-Z	-1,28	-0,40	0,73	0,54	1,10	1,65	1,26
		AE, in months,	-	4	6	19	22	-	-
		W/A-Z	1,11	-0,51	-0,86	-0,30	-0,21	-	-
		H/A-Z	0,43	0,20	-0,22	0,05	-0,89	-	-
		BMI/A-Z	-	-0,84	-0,98	-0,45	0,50	-	-
33	Male	HC/A-Z	2,16	0,68	0,89	1,02	1,42	-	-
		AE, in months,	-	6	8	14	19	-	-
		W/A-Z	-0,16	-0,10	0,06	-0,07	-0,21	-	-
		H/A-Z	-0,77	-1,18	-1,09	-2,36	-1,79	-	-
		BMI/A-Z	-	0,79	0,95	1,81	1,25	-	-
34	Male	HC/A-Z	1,11	0,45	0,56	0,27	0,63	-	-
		AE, in months,	-	8	17	31	-	-	-
		W/A-Z	-0,81	-0,63	-1,01	-1,54	-	-	-
		H/A-Z	0,04	0,13	-0,34	-0,51	-	-	-
		BMI/A-Z	-	-0,95	-1,15	-1,93	-	-	-
35	Male	HC/A-Z	-1,15	-1,4	-1,74	-1,98	-	-	-
		AE, in months,	-	7	-	-	-	-	-
		W/A-Z	-1,49	1,52	-	-	-	-	-
		H/A-Z	-1,38	-0,12	-	-	-	-	-
36	Female	BMI/A-Z	-	2,12	-	-	-	-	-

		HC/A-Z	-1,63	-0,01	-	-	-	-	-
		AE, in months,	-	5	7	12	15	18	21
		W/A-Z	-2,21	0,60	0,91	0,72	0,92	0,59	1,07
		H/A-Z	-1,76	-1,34	-0,29	0,15	-0,21	-0,61	-0,95
		BMI/A-Z	-	1,83	1,44	0,87	1,43	1,30	2,25
37	Female	HC/A-Z	-0,91	0,06	0,77	0,79	0,24	0,90	1,12
		AE, in months,	-	3	4	6	10	12	-
		W/A-Z	-0,12	0,52	0,90	1,37	1,23	1,04	-
		H/A-Z	0,94	-0,01	0,16	0,89	1,29	0,52	-
		BMI/A-Z	-	0,71	1,07	1,12	0,68	1,00	-
38	Male	HC/A-Z	0,26	1,88	1,26	1,44	0,30	1,02	-
		AE, in months,	-	5	8	12	18	-	-
		W/A-Z	0,17	0,17	-0,21	-0,39	0,35	-	-
		H/A-Z	2,58	0,28	0,01	-0,52	-0,16	-	-
		BMI/A-Z	-	0,01	-0,32	-0,14	0,62	-	-
39	Female	HC/A-Z	0,08	0,30	0,17	0,65	0,74	-	-
		AE, in months,	-	3	6	9	12	18	24
		W/A-Z	-2,44	-2,07	-1,61	-1,88	-1,70	-2,03	-2,41
		H/A-Z	-2,14	-1,83	-1,26	-0,75	-1,19	-1,15	-1,41
		BMI/A-Z	-	-1,39	-1,19	-2,01	-1,41	-1,91	-2,25
40	Female	HC/A-Z	-1,62	-1,25	-1,12	-0,61	-1,58	-1,70	-1,80
		AE, in months,	-	11	-	-	-	-	-
		W/A-Z	-1,07	-2,07	-	-	-	-	-
		H/A-Z	-1,60	-1,77	-	-	-	-	-
		BMI/A-Z	-	-1,41	-	-	-	-	-
41	Female	HC/A-Z	-0,37	-0,48	-	-	-	-	-

		AE, in months,	-	11	17	-	-	-	-
		W/A-Z	1,13	-0,46	-0,43	-	-	-	-
		H/A-Z	-0,41	-0,51	-0,05	-	-	-	-
		BMI/A-Z	-	-0,24	-0,58	-	-	-	-
42	Female	HC/A-Z	0,52	-0,31	-0,04	-	-	-	-
		AE, in months,	-	13	16	-	-	-	-
		W/A-Z	-0,18	1,82	1,66	-	-	-	-
		H/A-Z	0,35	-2,12	0,01	-	-	-	-
		BMI/A-Z	-	4,20	2,27	-	-	-	-
43	Female	HC/A-Z	0,09	2,00	1,87	-	-	-	-
		AE, in months,	-	12	-	-	-	-	-
		W/A-Z	-1,11	-0,84	-	-	-	-	-
		H/A-Z	-2,45	-3,51	-	-	-	-	-
		BMI/A-Z	-	1,86	-	-	-	-	-
44	Male	HC/A-Z	-1,15	-0,99	-	-	-	-	-
		AE, in months,	-	12	15	16	17	-	-
		W/A-Z	1,03	1,78	2,08	2,73	2,33	-	-
		H/A-Z	-	-0,84	0,33	0,50	1,27	-	-
		BMI/A-Z	-	3,05	2,61	3,40	2,28	-	-
45	Female	HC/A-Z	-0,71	-0,77	0,24	0,10	-0,46	-	-
		AE, in months,	-	10	13	23	-	-	-
		W/A-Z	0,19	-0,16	-0,30	-1,19	-	-	-
		H/A-Z	1,42	-3,33	0,01	-1,53	-	-	-
		BMI/A-Z	-	2,43	-0,42	-0,32	-	-	-
46	Male	HC/A-Z	1,29	0,26	0,50	0,66	-	-	-
		AE, in months,	-	2	4	5	7	13	32

		W/A-Z	0,12	0,07	0,13	-0,02	-0,03	-0,17	-0,33
		H/A-Z	-0,71	0,16	0,18	-0,17	-0,10	0,13	-0,37
		BMI/A-Z	-	-0,04	0,02	0,09	0,02	-0,36	-0,20
47	Female	HC/A-Z	-0,83	-0,01	-0,21	-0,81	-0,61	-0,20	-0,47
		AE, in months,	-	15	21	23	-	-	-
		W/A-Z	1,43	-0,59	-0,73	-1,29	-	-	-
		H/A-Z	-0,14	-1,40	-0,32	-1,03	-	-	-
		BMI/A-Z	-	0,32	-0,79	-0,96	-	-	-
48	Female	HC/A-Z	1,94	0,52	0,15	0,60	-	-	-
		AE, in months,	-	12	-	-	-	-	-
		W/A-Z	-0,55	-1,19	-	-	-	-	-
		H/A-Z	-2,28	-1,2	-	-	-	-	-
		BMI/A-Z	-	-0,65	-	-	-	-	-
49	Male	HC/A-Z	0,41	-0,82	-	-	-	-	-
		AE, in months,	-	12	-	-	-	-	-
		W/A-Z	0,70	1,74	-	-	-	-	-
		H/A-Z	-0,10	0,14	-	-	-	-	-
		BMI/A-Z	-	2,23	-	-	-	-	-
50	Male	HC/A-Z	1,43	1,31	-	-	-	-	-
		AE, in months,	-	12	-	-	-	-	-
		W/A-Z	0,04	1,62	-	-	-	-	-
		H/A-Z	0,43	-0,61	-	-	-	-	-
		BMI/A-Z	-	2,65	-	-	-	-	-
51	Male	HC/A-Z	-0,78	-0,58	-	-	-	-	-
		AE, in months,	-	13	22	-	-	-	-
52	Male	W/A-Z	-0,85	-0,76	-1,01	-	-	-	-

		H/A-Z	-0,92	-2,96	-2,36	-	-	-	-
		BMI/A-Z	-	1,40	0,73	-	-	-	-
		HC/A-Z	0,44	0,07	0,39	-	-	-	-
		AE, in months,	-	10	13	16	19	22	-
		W/A-Z	0,39	-1,72	-2,07	-2,61	-1,99	-1,73	-
		H/A-Z	-	-2,66	-2,22	-1,86	-2,06	-1,61	-
		BMI/A-Z	-	-0,18	-1,02	-2,08	-1,02	-1,04	-
53	Female	HC/A-Z	-	-1,65	-1,4	-1,71	-1,38	-1,46	-
		AE, in months,	-	7	9	11	14	25	37
		W/A-Z	1,41	0,34	0,43	0,59	0,43	0,63	0,24
		H/A-Z	-0,14	1,11	0,29	1,02	0,82	0,71	-0,03
		BMI/A-Z	-	-0,38	0,35	0,02	-0,06	0,25	0,35
54	Female	HC/A-Z	1,11	-0,67	0,61	1,66	1,02	-	1,18
		AE, in months,	-	20	30	-	-	-	-
		W/A-Z	0,05	-0,86	-1,14	-	-	-	-
		H/A-Z	-1,35	-1,66	-1,55	-	-	-	-
		BMI/A-Z	-	0,25	-0,24	-	-	-	-
55	Male	HC/A-Z	-0,78	-0,89	-0,91	-	-	-	-
		AE, in months,	-	19	25	-	-	-	-
		W/A-Z	0,76	0,09	0,89	-	-	-	-
		H/A-Z	0,43	-0,29	0,02	-	-	-	-
		BMI/A-Z	-	0,40	1,24	-	-	-	-
56	Male	HC/A-Z	1,33	-0,01	0,48	-	-	-	-
		AE, in months,	-	4	5	7	8	11	18
		W/A-Z	0,06	0,17	0,32	1,28	0,52	0,39	0,73
57	Female	H/A-Z	-1,21	-0,67	1,10	1,06	0,70	-0,20	0,82

		BMI/A-Z	-	0,73	-0,40	0,92	0,17	0,68	0,36
		HC/A-Z	2,00	-0,98	-2,85	-1,84	-2,30	-0,94	-0,64
		AE, in months,	-	12	-	-	-	-	-
		W/A-Z	-1,01	0,17	-	-	-	-	-
		H/A-Z	-0,41	-0,49	-	-	-	-	-
		BMI/A-Z	-	0,61	-	-	-	-	-
58	Female	HC/A-Z	-1,62	-0,38	-	-	-	-	-
		AE, in months,	-	19	26	31	38	-	-
		W/A-Z	0,25	-0,48	-0,25	0,11	-0,06	-	-
		H/A-Z	-0,57	-1,72	-0,89	-1,35	-0,66	-	-
		BMI/A-Z	-	0,82	0,43	1,40	0,50	-	-
59	Male	HC/A-Z	-0,27	-0,10	0,22	0,28	-0,09	-	-
		AE, in months,	-	9	11	14	18	20	37
		W/A-Z	-1,69	-1,32	-0,80	-0,97	-1,30	-0,96	-1,51
		H/A-Z	-1,74	-1,51	-1,16	-0,83	-0,75	-0,98	-1,12
		BMI/A-Z	-	-0,59	-0,16	-0,67	-1,21	-0,51	-1,25
60	Male	HC/A-Z	-2,00	-0,11	0,15	-0,45	0,37	0,90	-
		AE, in months,	-	3	5	22	34	41	--
		W/A-Z	-1,57	-0,20	-0,98	-1,06	-0,96	-0,78	-
		H/A-Z	-0,77	-0,09	-1,20	-1,08	-1,17	-1,69	-
		BMI/A-Z	-	-0,20	-0,37	-0,56	-0,35	0,49	-
61	Male	HC/A-Z	-0,78	0,12	-0,29	0,00	-	-0,25	-
		AE, in months,	-	2	4	6	19		-
		W/A-Z	1,88	0,25	1,25	1,52	1,16	-	-
		H/A-Z	0,43	0,56	0,29	0,90	1,18	-	-
62	Male	BMI/A-Z	-	-0,09	1,44	1,31	0,68	-	-

		HC/A-Z	1,72	0,56	0,11	1,21	0,29	-	-
		AE, in months,	-	20	23	40	-	-	-
		W/A-Z	-0,27	-1,50	-1,36	-1,97	-	-	-
		H/A-Z	0,49	-2,18	-1,48	-1,64	-	-	-
		BMI/A-Z	-	-0,18	-0,63	-1,36	-	-	-
63	Female	HC/A-Z	1,94	0,25	-0,05	0,41	-	-	-
		AE, in months,	-	1	11	35	-	-	-
		W/A-Z	0,05	-0,32	0,37	0,19	-	-	-
		H/A-Z	0,32	0,39	-0,04	-0,08	-	-	-
		BMI/A-Z	-	-0,76	0,57	0,31	-	-	-
64	Male	HC/A-Z	-0,63	2,20	-0,60	-1,01	-	-	-
		AE, in months,	-	5	6	8	11	15	
		W/A-Z	-0,62	-2,25	-2,27	-1,59	-1,59	-1,93	-
		H/A-Z	-1,48	-3,48	-2,31	-2,45	-2,81	-3,12	-
		BMI/A-Z	-	-0,22	-1,22	-0,18	0,14	-0,01	-
65	Male	HC/A-Z	-2,77	-6,83	-6,02	-5,15	-5,1	-5,7	-
		AE, in months,	-	12	-	-	-	-	-
		W/A-Z	3,41	0,12	-	-	-	-	-
		H/A-Z	1,37	-0,27	-	-	-	-	-
		BMI/A-Z	-	0,42	-	-	-	-	-
66	Male	HC/A-Z	2,70	0,29	-	-	-	-	-
		AE, in months,	-	21	26	-	-	-	-
		W/A-Z	-0,62	-0,29	-0,86	-	-	-	-
		H/A-Z	-0,41	1,07	-0,17	-	-	-	-
		BMI/A-Z	-	-1,37	-1,17	-	-	-	-
67	Female	HC/A-Z	-0,37	0,13	0,02	-	-	-	-

		AE, in months,	-	1	5	8	10	13	15
		W/A-Z	-0,52	-1,84	-0,47	-0,02	-0,01	-0,13	-0,06
		H/A-Z	-0,77	-1,87	-0,89	0,07	0,16	0,10	0,22
		BMI/A-Z	-	-1,28	0,07	-0,06	-0,12	-0,24	-0,25
68	Male	HC/A-Z	-0,78	-0,29	-0,08	0,83	0,39	0,37	0,10
		AE, in months,	-	12	-	-	-	-	-
		W/A-Z	0,67	-1,09	-	-	-	-	-
		H/A-Z	-0,17	-1,78	-	-	-	-	-
		BMI/A-Z	-	0,04	-	-	-	-	-
69	Male	HC/A-Z	2,46	2,24	-	-	-	-	-
		AE, in months,	-	2	5	6	10	30	33
		W/A-Z	0,13	-0,70	-0,73	-0,32	-0,26	-0,35	-0,17
		H/A-Z	1,04	-0,37	0,65	0,53	0,19	-0,06	-0,11
		BMI/A-Z	-	-0,69	-1,50	-0,85	-0,53	-0,53	-0,20
70	Female	HC/A-Z	1,16	-1,67	-1,31	-0,94	1,09	-0,50	-0,31
		AE, in months,	-	2	6	10	12	18	26
		W/A-Z	1,28	1,56	2,65	2,68	2,62	1,77	1,13
		H/A-Z	1,96	0,09	3,67	1,41	1,64	0,01	1,23
		BMI/A-Z	-	2,05	0,80	2,43	2,22	2,38	0,53
71	Male	HC/A-Z	2,56	3,00	3,64	2,96	3,23	2,64	2,83

W/A-Z, H/A-Z, BMI/A-Z, HC/A-Z: Z-score Weight, Height, BMI (body mass index) and HC (head circumference) for Age, respectively.
AE: Age at evaluation, in months.
